# Radiological and Functional Outcome of Displaced Colles’ Fracture Managed with Closed Reduction and Percutaneous Pinning: A Prospective Study

**DOI:** 10.7759/cureus.960

**Published:** 2017-01-06

**Authors:** Sagar Panthi, Kishor Khatri, Krishna Kharel, Subin Byanjankar, Jay R Sharma, Rahul Shrestha, Raju Vaishya, Amit Kumar Agarwal, Vipul Vijay

**Affiliations:** 1 Orthopaedics and Trauma Surgeon, Nepalgunj Medical College and Teaching Hospital; 2 Orthopaedics and Trauma Surgeon, Lumbini Zonal Hospital; 3 Orthopaedics, Tilottama Hospital; 4 Ortthopaedics, Lumbini Medical College, Nepal; 5 Orthoapedics, Anandaban Hospital, Lalitpur; 6 Orthopaedics, Lumbini Medical College, Nepal; 7 Orthopaedics, Indraprastha Apollo Hospitals

**Keywords:** colles fracture, percutaneous pinning, functional outcome

## Abstract

Background: Displaced Colles’ fractures are treated by manipulation and below elbow cast application. Malunion is a common complication, resulting in pain, mid-carpal instability, and post-traumatic arthritis. Fracture stabilization by percutaneous pinning is a simple, minimally invasive technique that helps prevent displacement of the fracture, thereby minimizing complications. This study aims to assess the amount of collapse after closed manipulation and percutaneous pinning with Kirschner wires (K-wires) and its correlation with the functional outcome of the wrist after union.

Methods: A prospective study was conducted from May 2015 to May 2016 in a tertiary orthopedic center. Ninety patients (60 females, 30 males) with an average age of 54.93 years with Type II fractures underwent closed manipulation and percutaneous pinning with crossed K-wires as the primary procedure. Serial radiographs were taken to document the amount of collapse. The functional outcome was assessed using the Cooney Wrist Score.

Results: At the final follow-up at six months, the collapse in the mean dorsal angle was 0.94 and mean ulnar variance was 0.51. Functionally, 48 patients (53.33%) had an excellent outcome, 36 patients (40%) had a good outcome, and six patients (6.67%) had a fair outcome.

Conclusions: Displaced Colles’ fractures should be reduced and stabilized with percutaneous K-wires to achieve an excellent functional outcome.

## Introduction

Colles’ fracture is an extra-articular distal radius fracture described by Abraham Colles in 1814. Colles’ fracture is a common fracture presentation in the orthopedic emergency department. It commonly affects the elderly female population. There is a direct relationship between osteoporosis and Colles’ fracture. Colles gave a description of a fracture of the distal radius, that is, within 2.5 cm above the wrist joint line, dorsally angulated and displaced, radially angulated and displaced, impacted and supinated, with or without distal radio-ulnar joint disruption [1]. Fractures of the distal radius were considered uncomplicated injuries in the past. Initially, Abraham Colles treated these fractures when there was no radiography, aseptic surgery, or anesthesia, and the amount of disability following malunion was accepted. Malunion results in pain, mid-carpal instability, and post-traumatic arthritis [2-3].

The degree of disability after a fracture of the distal radius has been shown to correlate with the amount of residual deformity [4]. Permanent loss of the palmar angle and radial shortening of the distal radius are associated with persisting wrist pain [5]. Treatment has changed over time as it has become more of a concern because of occupational disability and the need for prolonged care in previously independent elderly individuals [6]. After manipulation and plaster cast application, many of these fractures were displaced [7-8]. They reduce initially well, but there is a frequent loss of reduction as cast immobilization is a relatively inefficient means of stabilization [9]. Stable fractures can be managed conservatively by plaster cast immobilization alone with good to excellent anatomical and functional results [10]. Unstable fractures need some form of operative intervention.

Various methods for minimizing the loss of reduction in unstable fractures of the distal radius have been described. These include percutaneous pinning of the distal fragment, immobilization with pins incorporated in the plaster, external skeletal fixation, limited open reduction with or without bone grafting or bone substitutes, and extensive open reduction and internal fixation [11-14]. Percutaneous pinning has been recommended as a  simple way of providing additional stability as compared to immobilization in a cast which is unstable. In elderly or severely osteoporotic patients with a comminuted fracture, the technique of percutaneous pinning gives less favorable results and is therefore considered to be inappropriate [15].  

The aim of this prospective study was to assess the functional outcome and amount of collapse in patients with Colles’ fractures managed by the closed manipulation and percutaneous Kirschner wire (K-wire) fixation. Approval for this multicenter prospective study was granted by the Ethical Committee of Kathmandu Medical College, Nepal (protocol #286).

## Materials and methods

A prospective study was conducted in a tertiary care hospital from the period extending from May 2015 to May 2016. Ninety patients underwent closed manipulation and percutaneous pinning with crossed K-wires as the primary procedure. Cases presenting to emergency and outpatient department fulfilling the inclusion criteria were selected for the study (Table 1). Exclusion criteria were also described for careful selection of the cases.

**Table 1:** Inclusion and Exclusion Criteria

Inclusion Criteria	Exclusion Criteria
Displaced/impacted distal radial fracture Colles’ (Frykman Type I and II; Universal Type II)	Age less than 40 years and more than 70 years
Men and women between 40 to 70 years of age	Undisplaced fracture
	Open fracture
	Fractures associated with nerve, vessels, and tendon injury
	Bilateral fracture of wrist
	Previous distal radius fracture in the contralateral side
	Involvement of inflammatory disease in the opposite wrist, such as rheumatoid arthritis
	Grossly comminuted fracture
	Fracture presenting after one week of trauma

Informed consent was obtained from all the patients included in the study. Fracture displacement was characterized as displaced when there was dorsal angulation of > 10° and positive ulnar variance of > 3 mm. An acceptable reduction following closed reduction was a fracture with dorsal angulation of ≤ 0° and an ulnar variance of ≤ 3 mm. Radiographic measurements were made using a goniometer [1]. 

**Figure 1:**
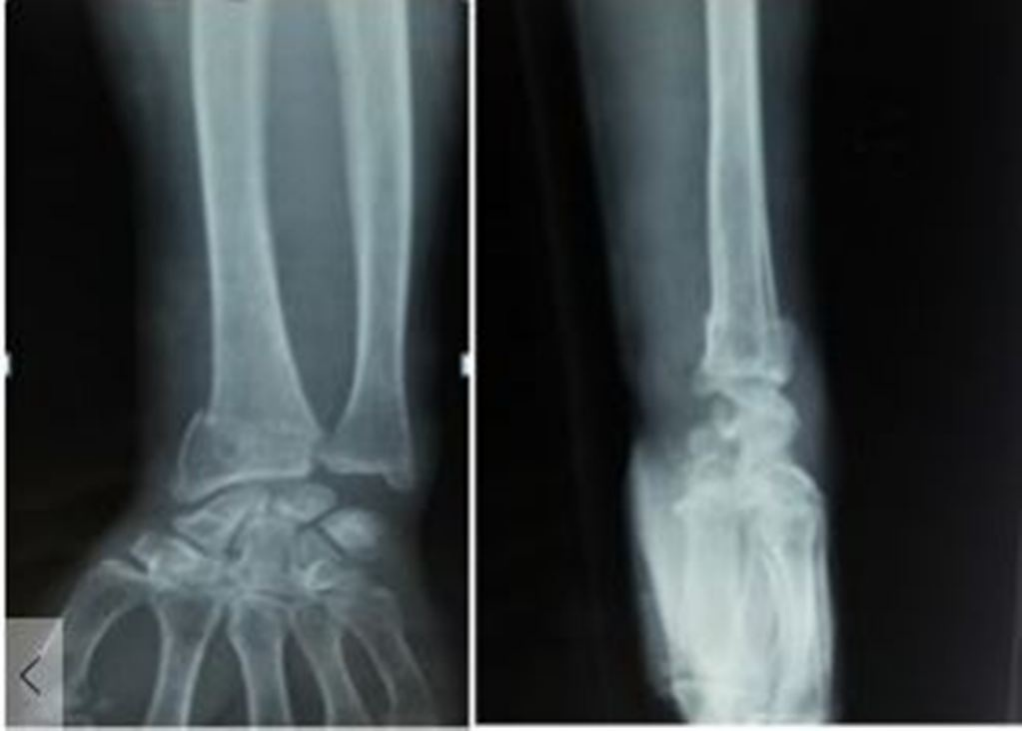
Radiographs anteroposterior and lateral view showing fracture of distal radius

A complete clinical history and associated comorbidities were recorded. A thorough physical examination was done in every case. The injured wrist was examined to identify the deformity and status of the nerve, vessels, and tendons. The contralateral wrist was also examined to rule out previous fractures and involvement of inflammatory disease. Anteroposterior (AP) and lateral radiographs were taken for assessing a distal radius fracture. AP view was taken with the shoulder in 90 degrees of abduction, and the elbow was flexed to 90 degrees with the wrist and forearm in a neutral position. The lateral view was taken with the shoulder adducted and the elbow flexed to 90 degrees with the hand positioned in the same plane as the humerus (Figure 1). Blood investigations required for pre-anaesthetic evaluation were carried out.

All procedures were carried out in the operation theater under general anesthesia or regional anesthesia. The upper extremity was prepared and draped free from the elbow. The surgeon and assistant were gowned and gloved. To allow easier access for the C-arm of the image intensifier, a hand table was used to support the limb. The reduction was accomplished by applying traction on the thumb and the other three lateral digits to distract the fracture while countertraction was applied proximal to the elbow by an assistant. The traction on the thumb was sustained while the contralateral hand was used to restore the normal volar tilt once distraction of the fracture was adequate.

The reduction was then evaluated in the anteroposterior and lateral planes with the image intensifier. Scrutiny towards the apposition and alignment of the volar surface of the fracture was one key feature in assessing the reduction. Once the length and the dorsal angle of the radius were restored, the fracture was fixed by two crossed 1.8 mm smooth K-wires, inserted percutaneously with a power drill.

The first K-wire was inserted at the tip of the radial styloid process just dorsal to the first extensor canal, in the anatomical snuff box proximal to the radial artery, aiming to cross the fracture line in both planes under image intensifier control. This requires about a 45-degree angle with the long axis of the radius on the anteroposterior view and aiming the wire 10 degrees dorsally on the lateral view. The second K-wire was inserted into the dorsal ulnar corner of the distal part of the radius between the fourth and fifth extensor canals. The correct line of aim that was required to cross the fracture was about 45 degrees on the anteroposterior view and 30 degrees dorsally on the lateral view.

**Figure 2:**
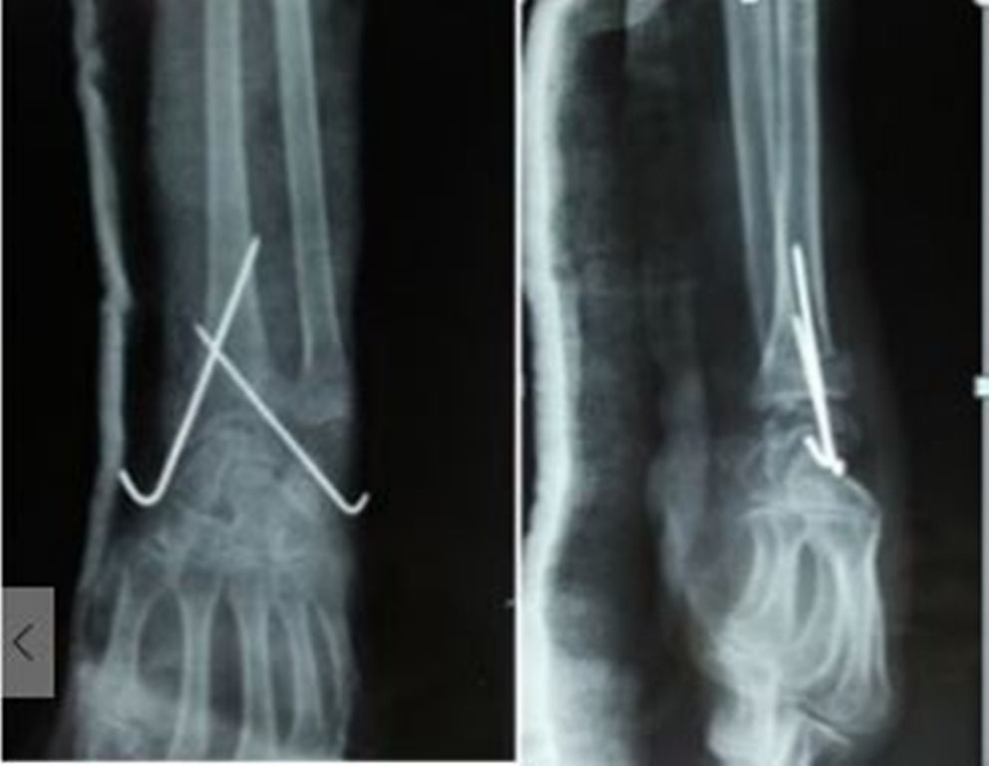
Post-reduction anteroposterior and lateral radiographs showing good reduction

Both K-wires are advanced just to penetrate the cortex of the proximal fragment. The accuracy of the reduction and the placement of the K-wires was again assessed with the image intensifier. The stability was finally evaluated by performing flexion and extension of the wrist under fluoroscopy. Both K-wires were then cut above the skin after bending them. Sterile gauze was placed over each pin site, and a padded short arm dorsal plaster slab was applied. The patient was discharged from the hospital following anteroposterior and lateral radiographs of the wrist after the operation (Figure 2). The patients were instructed to actively mobilize their fingers, elbow, and shoulder joints from the first postoperative day.

On the first follow-up, the plaster slab was discarded, and a forearm brace was applied. The patients were instructed to clean the pin sites with a spirit swab four to six times a day. At three weeks, radiographs were taken and assessed. At six weeks, all fractures had a radiological union and the K-wires were removed. Intermittent active motion of the wrist was then begun. Patients were encouraged to resume wrist movements. Unlimited activities were allowed from the third month. Patients were seen at six months for a final clinical and functional assessment.

Radiographic measurements of the postoperative dorsal angle and the ulnar variance were recorded and compared with the radiographs taken at the final assessment at six months to document the amount of collapse. We followed the method described by WP Cooney for functional evaluation (modified from the Green and O’Brien score). A final clinical and functional assessment was made using Cooney Wrist Score at six months (Table 2) [16].

**Table 2:** Cooney Wrist Score Table showing the Cooney Wrist score used for the assessment and functional evaluation of the wrist at final outcome. Excellent: ≥ 95 points; Good: ≥ 75 points; Fair: ≥ 60 points; Poor: < 60 points DF: dorsiflexion; PF: palmar flexion

Functional Evaluation	Total Points		Points
Pain	25	No pain	25
		Mild occasional	20
		Moderate tolerable	15
		Severe to intolerable	0
Functional status	25	Return to regular employment	25
		Restricted employment	20
		Able to work, unemployment	15
		Unable to work because of pain	0
Range of motion (Percentage of normal side)	Percentage of normal side	100%	25
		75 – 100%	15
		50 – 75%	10
		25-50%	5
		0-25%	0
	DF-PF arcs of injured wrist	120 degrees or more	25
		90 – 120 degrees	15
		60 – 90 degrees	10
		30 – 60 degrees	5
		30 degrees or less	0
Grip strength (Percentage of normal side)	25	100%	25
		75% - 100%	15
		50 – 75%	10
		25 – 50%	5
		0 – 25%	0

## Results

Ninety patients with Type II fractures underwent closed manipulation and percutaneous pinning with crossed K-wires as the primary procedure. Among the 90 patients, there were 60 female and 30 male patients. Their ages ranged from 42 to 70 years. The average age was 54.93 years. The right wrist was involved in 30 patients, whereas the left wrist was involved in 60 patients. Falling on an outstretched hand was the commonest mode of injury. 

The mean pre-reduction dorsal angulation and ulnar variance were 22.33 degrees and 3.66 mm, respectively. After surgery, the mean dorsal angulation and ulnar variance were -6.87 degrees and 1.17 mm, respectively. At six months follow-up, dorsal angulation was -5.93 degrees and the ulnar variance was 1.60 mm (Table 3).

**Table 3:** Variations in the Dorsal Angle and Ulnar Variance

	Mean	Std. Deviation	Range
Pre-reduction dorsal angle	22.33	7.743	12 - 25
Pre-reduction ulnar variance	3.66	0.628	3 - 5
Postoperative dorsal angle	-6.87	1.17	-11 to -3
Postoperative ulnar variance	1.17	0.632	0 - 2
Dorsal angle at 6 months	-5.93	2.756	-10 to 0
Ulnar variance at 6 months	1.60	0.594	1 - 3

Changes in the mean dorsal angulation and ulnar variance after surgery and at the six-month follow-up were -0.94 degrees and -0.51 mm, respectively (Table 4).

**Table 4:** Comparison of Changes in the Dorsal Angle and Ulnar Variance at the Postoperative Period and Six Months

	Mean	Std. Deviation	Range
Change in dorsal angle postop vs six months	-0.94	0.582	-4 - 0
Change in ulnar variance postop vs six months	-0.51	0.683	-2 - 0

The presentation of the patient before the operation ranged from 0-11 days (average: 1.84 days). The earlier presented fracture reduction was easier. The postoperative hospital stay ranged from one to 12 days. The average hospital stay was 1.66 days. Most patients were discharged the following morning after the surgery. The associated medical co-morbidities in some patients caused delays in their operative intervention and their discharge from the hospital.

Radiographic measurements were made using a goniometer to assess the amount of collapse. The preoperative mean dorsal angle and the mean ulnar variance were 22.33 and 3.66, respectively. Following surgical correction, the mean dorsal angle and ulnar variance were -6.87 and 1.17, respectively. The amount of collapse measured at the six-month final assessment in the mean dorsal angle and ulnar variance was 0.94 and 0.51, respectively (Figures 5-7). The data was analyzed using Statistical Package for Social Sciences (SPSS), version 16.0 (IBM SPSS Statistics, Armonk, NY). Means and standard deviations were calculated, and unpaired t-test was used to compare them. Using the Cooney Wrist Score, a clinical and functional assessment was made at six months. Forty-eight (53.33%) patients were found to have an excellent outcome, 36 (40%) had a good outcome, and six (6.67%) had a fair outcome. Six patients developed superficial pin site infection, which resolved with a course of antibiotics.

**Figure 3:**
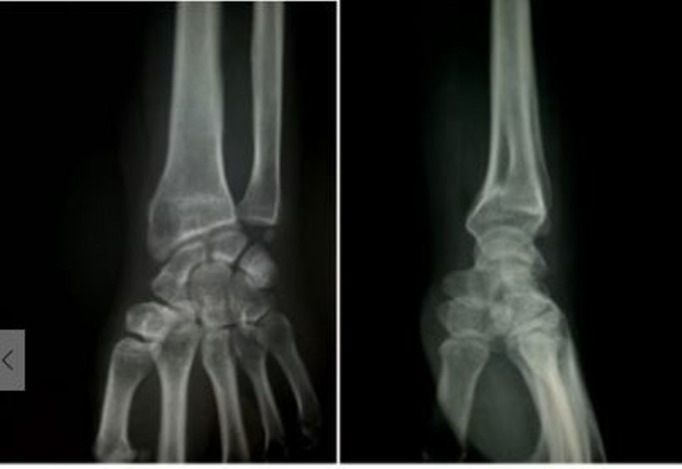
Anteroposterior and lateral x-ray of wrist after six months follow-up showing good union

**Figure 4:**
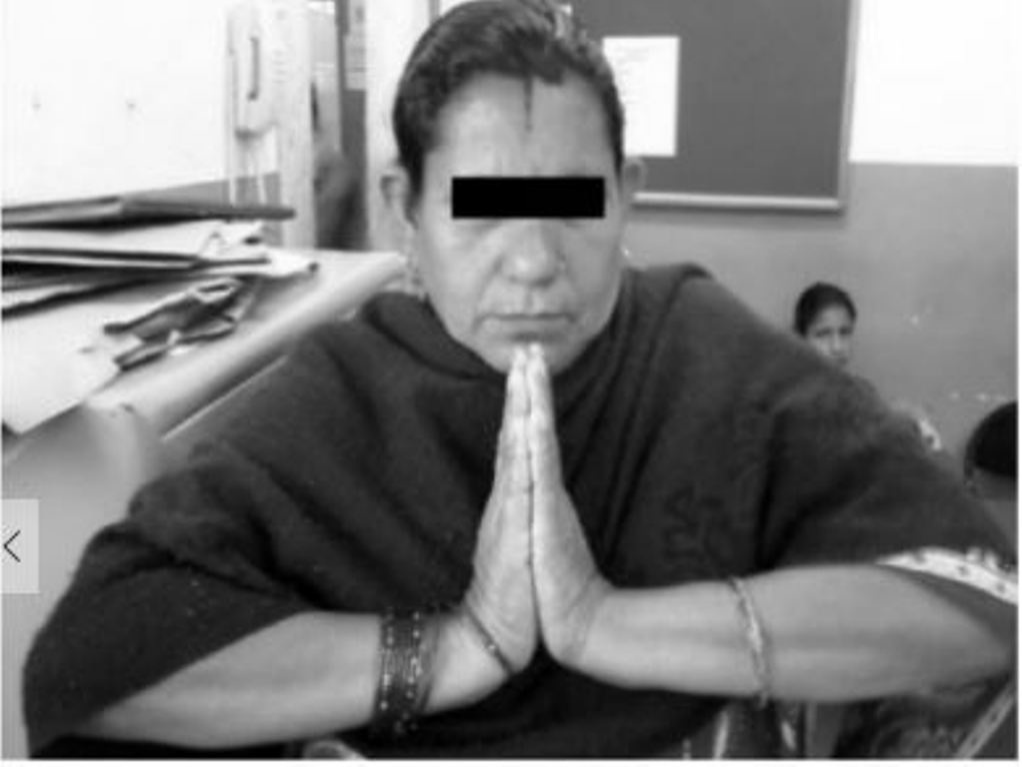
Clinical picture showing good dorsiflexion at final follow-up

**Figure 5:**
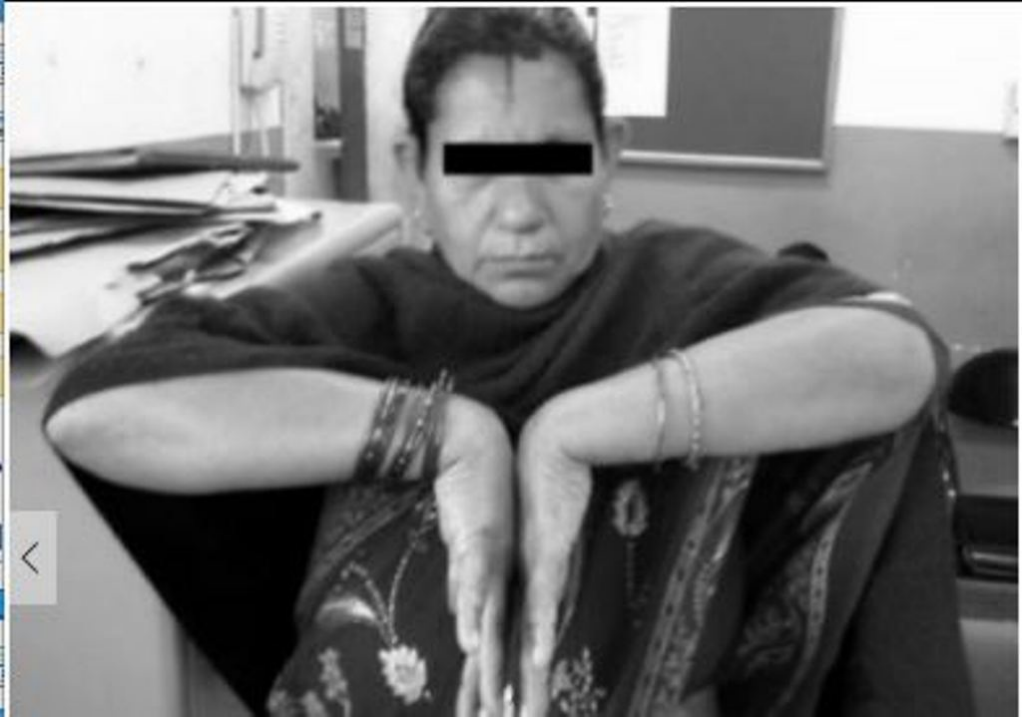
Clinical picture showing good palmar flexion at final follow-up

## Discussion

Fractures of the distal radius are commonly encountered in orthopedic practice. Several studies have suggested that there is a direct relationship between the anatomical reduction and functional outcome. However, older patients with lower functional demands do relatively well in spite of obvious deformity [17-22]. Closed manipulation can easily achieve the anatomical reduction, but there is still no agreement about the most appropriate way of maintaining the reduction in unstable fractures.

Colles advocated plaster cast stabilization to prevent deformity. There is considerable evidence that re-displacement is common and cosmetic results are far from perfect [23-24]. Seventy percent of cases undergoing conservative treatment are associated with significant displacement [25]. Various methods of internal and external stabilization devices, such as external fixation, the Roger Anderson device, use of Rush® pins (Rush Pin, LLC, Meridian, MS), and plaster techniques, have come into vogue over the years to prevent displacement in unstable fractures. Their objectives are targeted at restoration and stabilization of the anatomy of the distal radius.

In our study, the patients underwent closed manipulation and stabilization by percutaneous pinning primarily with two K-wires. No manipulation was performed before the procedure. The fractures were reduced and stabilized under the same anesthesia. Kurup, et al. studied the late collapse of distal radius fractures after K-wire removal and its significance [26]. They found that the fractures did not suffer significant loss of reduction after removal of wires. Loss of dorsal tilt was 2.6 degrees and ulnar variance was 1.3 mm. There has been no functional correlation in their study and whether the collapse affects the function was questionable.

Excellent results were reported by Stein and Katz in their comparative study, which involved percutaneous pinning of distal radius fractures and casting alone [27]. They confirmed the decrease in the radial shortening, maintenance of the normal volar tilt, and superior range of motion with percutaneous pinning. Dixon, Allen, and Bannister documented that the radial shortening improved after manipulation and casting to less than 3 mm in 86% of patients (79/92) but was maintained in 48% (44/92) after three months [28]. They concluded that there was room for improvement in the treatment of this common fracture as there was a 73% risk of failure following manipulation and plaster cast fixation.

Azzopardi, et al. performed a prospective randomized study on 57 patients, older than 60 years of age with unstable, extra-articular fractures of the distal radius to compare the outcome of immobilization in a cast alone with closed reduction percutaneous pinning [29]. Patients treated by percutaneous pinning had a statistically significant improvement in dorsal angulation, radial length, and radial inclination at the one year period. Anatomical reduction, which is achieved by manipulation under anesthesia, is an integral part of the management of this fracture. After the anatomy is restored, the maintenance of the accomplishment has to be secure. Percutaneous pinning is an excellent technique [30]. The patients received their treatment under a single exposure to anesthesia. This helped decrease the morbidity. In patients that presented earlier, the reduction was easier to perform so early intervention is of help in the management.

In our study, the ends of the K-wires were bent and left outside the skin. Clancy cut the K-wires and allowed the ends to retract subcutaneously [14]. The patients required anesthesia to remove the K-wires later. Our patients were taught pin site cleaning, which they performed four to six times a day. It helped reduce the pin site complications, and we encountered only two cases of superficial tract infection that subsided with a course of antibiotics. The advantage of the ends of the K-wires being outside was their easy removal. 

## Conclusions

Colles’ fracture is more common in the elderly female population. Closed reduction and percutaneous pinning is an excellent technique for stabilization of displaced Colles’ type fracture. After closed reduction and percutaneous pinning, better restoration and maintenance of dorsal angulation and ulnar variance were observed. Unstable Colles fractures must be reduced acceptably. The functional outcome was excellent in 48 (53.33%) patients, good in 36 (40%), and fair in six (6.67%).
